# Impact of deep learning image reconstruction algorithms on CT radiomic features in patients with liver tumors

**DOI:** 10.3389/fonc.2023.1167745

**Published:** 2023-04-05

**Authors:** Gongbo Xue, Hongyan Liu, Xiaoyi Cai, Zhen Zhang, Shuai Zhang, Ling Liu, Bin Hu, Guohua Wang

**Affiliations:** ^1^ Department of Radiology, Qingdao Municipal Hospital, Qingdao, China; ^2^ Graduate School, Dalian Medical University, Dalian, China; ^3^ CT Imaging Research Center, GE Healthcare China, Shanghai, China; ^4^ Department of Radiology, The Affiliated Hospital of Qingdao University, Qingdao, China

**Keywords:** computed tomography, texture analysis, liver, deep learning, image reconstruction

## Abstract

**Objective:**

To evaluate the impact of deep learning image reconstruction (DLIR) and adaptive statistical iterative reconstruction-Veo (ASIR-V) on abdominal CT radiomic features acquired in portal venous phase in liver tumor patients.

**Methods:**

Sixty patients with liver tumors who underwent contrast-enhanced abdominal CT were retrospectively enrolled. Six groups including filtered back projection (FBP), ASIR-V (30%, 70%) and DLIR at low (DLIR-L), medium (DLIR-M and high (DLIR-H), were reconstructed using portal venous phase data. CT-based radiomic features (first-order, texture and wavelet features) were extracted from 2D and 3D liver tumors, peritumor and liver parenchyma. All features were analyzed for comparison. *P* < 0.05 indicated statistically different. The consistency of 3D lesion feature extraction was assessed by calculating intraclass correlation coefficient (ICC).

**Results:**

Different reconstruction algorithms influenced most radiomic features. The percentages of first-order, texture and wavelet features without statistical difference among 2D and 3D lesions, peritumor and liver parenchyma for all six groups were 27.78% (5/18), 5.33% (4/75) and 5.56% (1/18), respectively (all *p* > 0.05), and they decreased while the level of reconstruction strengthened for both ASIR-V and DLIR. Compared with FBP, the features of ASIR-V30% and 70% without statistical difference decreased from 71.31% to 23.95%, and DLIR-L, DLIR-M, and DLIR-H decreased from 31.65% to 27.11% and 23.73%. Among texture features, unaffected features of peritumor were larger than those of lesions and liver parenchyma, and unaffected 3D lesions features were larger than those of 2D lesions. The consistency of 3D lesion first-order features was excellent, with intra- and inter-observer ICCs ranging from 0.891 to 0.999 and 0.880 to 0.998.

**Conclusions:**

Both ASIR-V and DLIR algorithms with different strengths influenced the radiomic features of abdominal CT images in portal venous phase, and the influences aggravated as reconstruction strength increased.

## 1 Introduction

Quantitative imaging has been the state-of-the-art approach in oncologic medical images (1). Among the methods to explore CT quantification, CT texture analysis (CTTA), as a non-invasive one, can quantitatively assess the ultrastructure of tissues by analyzing the pixel grayscale distribution of CT images and reflect their heterogeneity in the microscopic environment (2). Although CTTA was demonstrated to own high clinical research and application value, especially for application in patients with tumor (3–6), most CTTAs so far had been retrospective studies, and differences in CT scanning equipment, CT acquisition parameters (reconstruction section thickness, algorithms, and kernel, etc.) could lead to differences in CT texture features (7–9). Therefore, it is crucial to investigate the effects of variances reconstruction algorithms on CT images for the credibility of clinical applications of CTTA.

Current research involving CTTA focused on the quantitative analysis of images reconstructed by applying the filtered back projection (FBP) algorithm. However, FBP did not meet the current diagnostic needs under low radiation doses, such as low-contrast lesions (10). Nowadays, to satisfy the demand of low dose and high image quality in CT images, various manufacturers had introduced iterative algorithms, such as adaptive statistical iterative reconstruction-Veo (ASIR-V, GE Healthcare) (11). Although the application of the iterative algorithm could indirectly reduce the radiation dose to some extent by reducing the noise, it had been shown that the algorithm could alter the noise texture of the image, making the image look unnatural, especially when the strength of the iteration increased (12). With the continuous development of artificial intelligence in the medical field, deep learning-based reconstruction algorithms have emerged, such as the deep learning image reconstruction (DLIR) algorithm developed by GE Healthcare with three levels (DLIR-L, DLIR-M, DLIR-H), which can be selected by users according to different clinical scenarios. It was shown that this algorithm could reduce CT images’ noise without affecting anatomical and pathological structures and without changing the noise texture (13).

Different iterative algorithms and various iteration strengths had been verified to have an impact on the extraction of radiomic features (14–16). However, recent studies of deep learning image reconstruction algorithms had mainly explored their impact on image quality and radiation dose (17, 18), the impact of DLIR algorithms on radiomic features was still not well known. The purpose of this study was to evaluate the impact of different strengths of ASIR-V and DLIR compared to FBP on the CT imaging radiomic features of patients with liver tumors and to verify their stability in the quantitative analysis of contrast-enhanced CT.

## 2 Materials and methods

### 2.1 Clinical data

**Figure 1:**
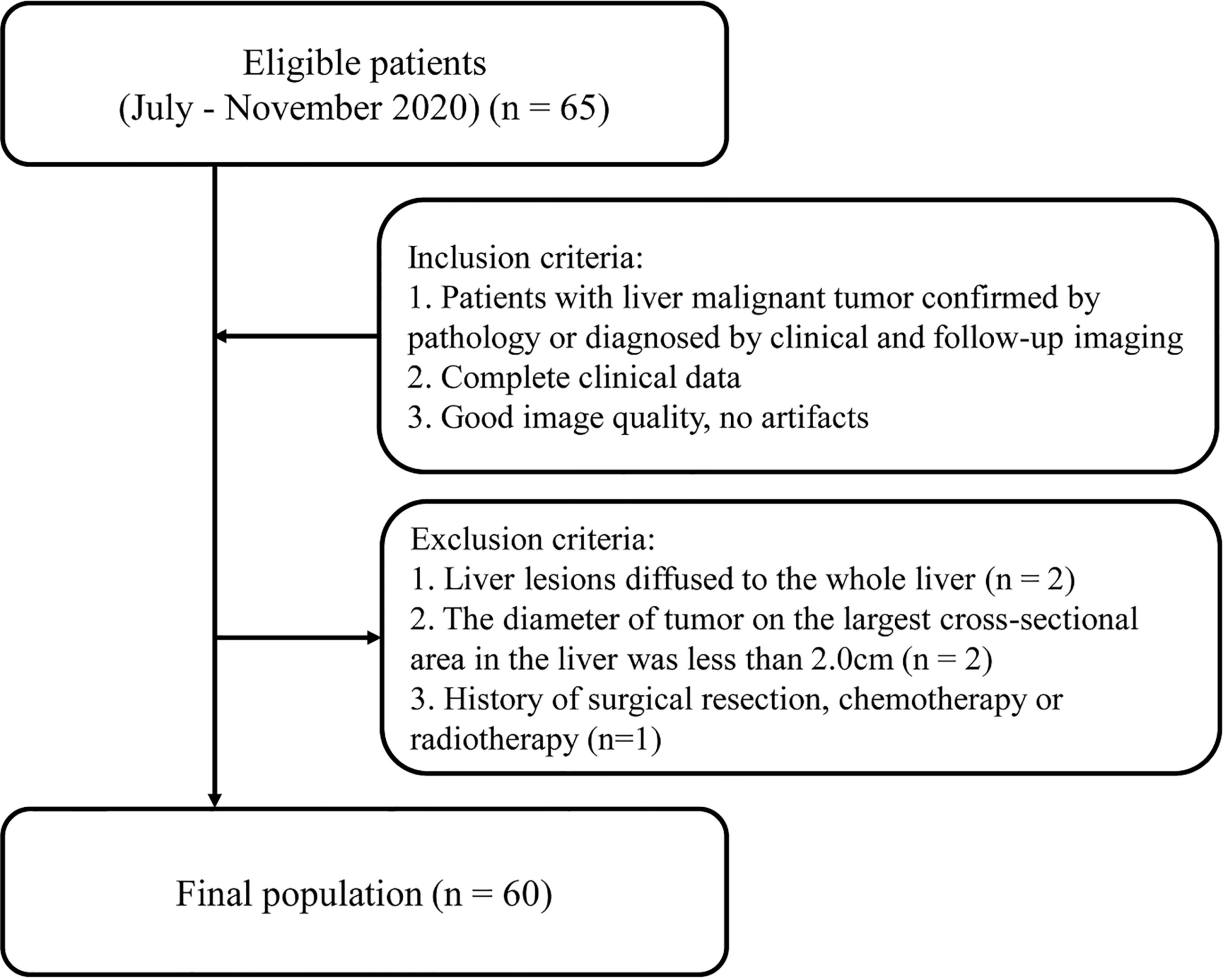
Flowchart of patient recruitment.

Adult patients with liver tumors who underwent abdominal CT enhancement examination in Qingdao Municipal Hospital from July to November 2020 were retrospectively collected. Inclusion criteria were as follows: (1) Patients with liver malignant tumor confirmed by pathology or diagnosed by clinical and follow-up imaging; (2) Completed clinical data; (3) Good image quality, no artifacts. Exclusion criteria included the following: (1) Liver lesions diffused to the whole liver; (2) The diameter of tumor on the largest cross-sectional area in the liver was less than 2.0 cm (7); (3) History of surgical resection, chemotherapy or radiotherapy (**Figure 1**). The study was approved by the Ethics Committee of Qingdao Municipal Hospital affiliated to Qingdao University, and the informed consent of each patient was exempted.

### 2.2 CT examination method

All participants underwent abdominal contrast-enhanced CT on a 256-slice spiral CT (Revolution CT, GE Healthcare, Milwaukee, USA) in supine position, with arms above head to prevent artifacts. During scanning, patients cautiously followed the breath-hold instructions and the scanning range was from the diaphragm top to the bottom of the liver. The scanning parameters were as follows: tube voltage 120kV, tube current automatic modulation range 250~500mA, noise index 8.5, pitch 0.992:1, rotational speed 0.8s, scanning slice thickness 5mm. The IV contrast agent used was Ioversol (320mgI/ml, Jiangsu Hengrui Pharmaceuticals Co., Ltd., China). Injection of contrast agent through the elbow vein using a high-pressure syringe (DUAL SHOT alpha7, Nemoto, Japan) at a flow rate of 2.2ml/s, and the weight-based contrast dosing protocol was 1.5ml/kg. The arterial phase, portal venous phase and delay phase were scanned with a delay of 30s, 60s and 120s after contrast injection, respectively.

### 2.3 CT texture analysis

**Figure 2:**
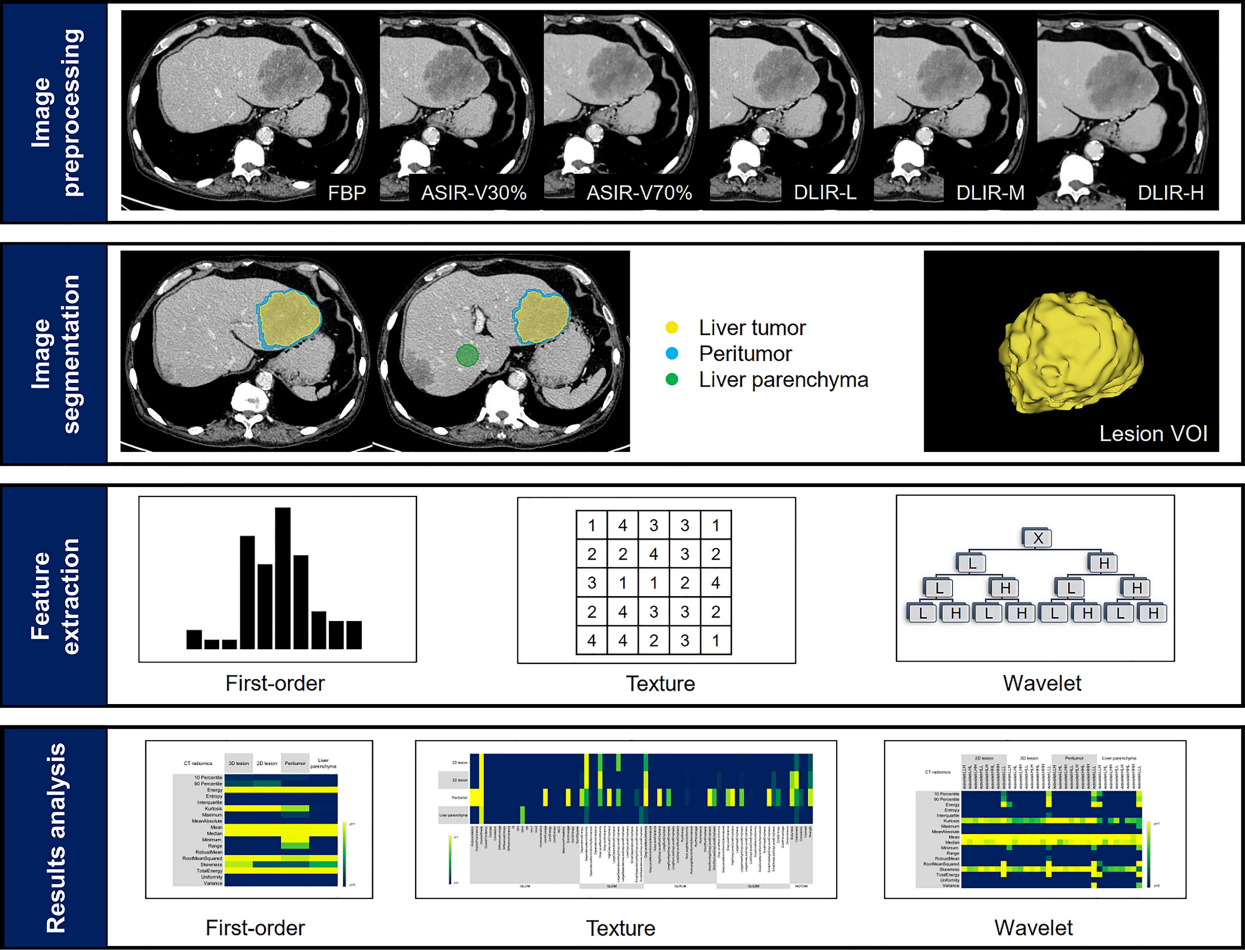
Study workflow. The workflow presented a summary of image reconstruction, target lesions annotation and dilation, feature extraction and results analysis.

The study workflow of CT texture analysis was shown in **Figure 2**. Specific steps were as follows.

#### 2.3.1 Image reconstruction

Six groups of images, i.e., FBP, ASIR-V30%, ASIR-V70%, DLIR-L, DLIR-M and DLIR-H, were reconstructed using the original scan datasets in portal venous phase. All CT scan images were reconstructed with 1.25mm slice thickness and transmitted to the picture archiving and communication systems (PACS) for anonymous processing and DICOM format image export.

#### 2.3.2 ROI segmentation

DICOM images were transferred from PACS to 3D Slicer software (version 4.11, https://www.slicer.org). Regions of interests (ROI) were segmented by two radiologists (A, with three years of experience in abdominal radiology and B, with two years of experience in abdominal radiology). Radiologist A manually segmented ROI on the tumor lesion on the FBP images: (1) 3D tumor was delineated layer by layer on the axial images; (2) 2D tumor was the largest cross-sectional area of the tumor. Radiologist B performed the same 3D tumor segmentation on the FBP image again. For multiple lesions, the largest one was selected for segmentation, and the tumor lesions were segmented to avoid adjacent vessels and intrahepatic bile ducts. All ROIs were segmented to obtain the 3D volume of interest (VOI) by 3D slicer. Radiologist A segmented the 3D tumor focal axial images again after 1 month.

**Figure 3:**
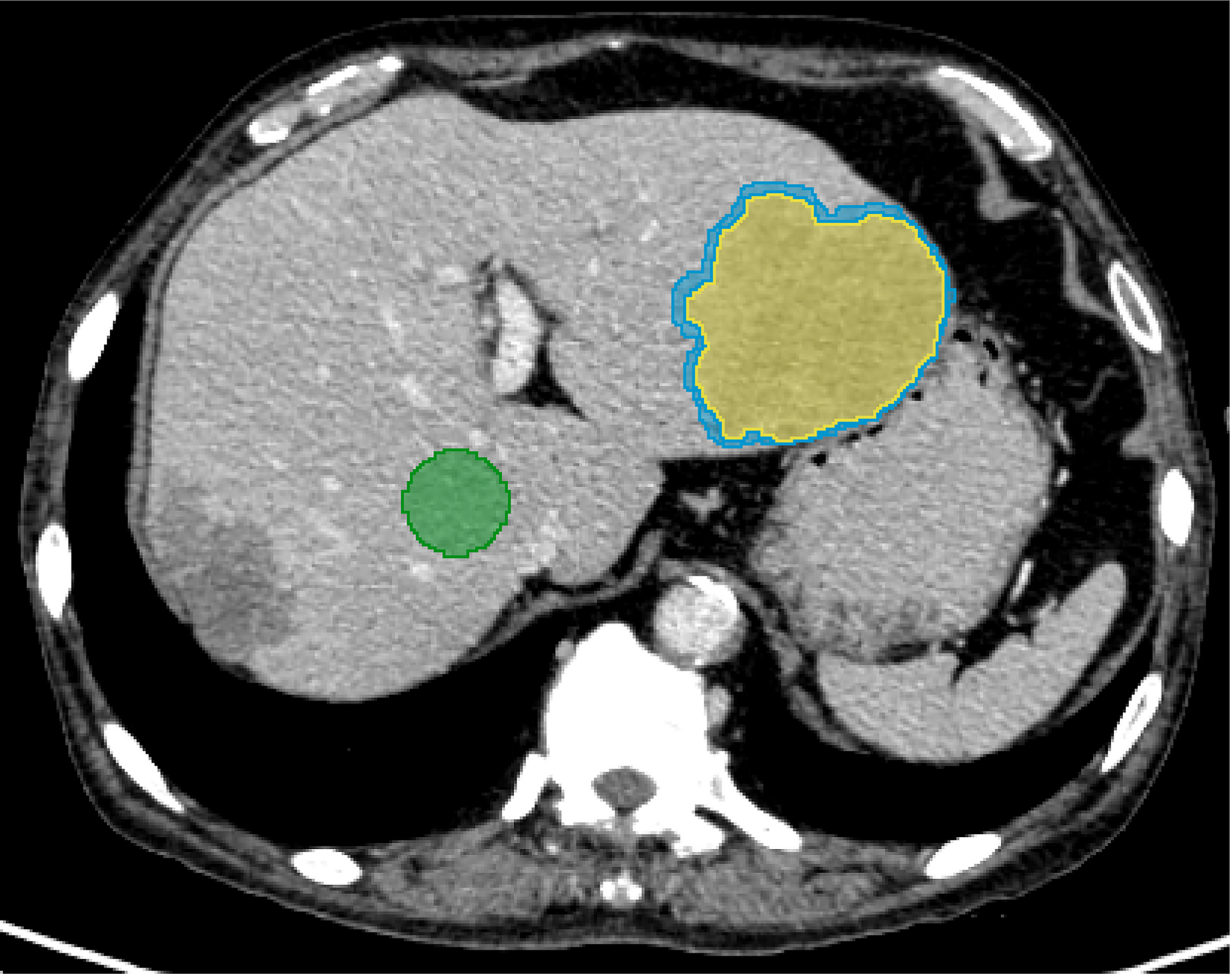
A 67-year-old male diagnosed with prostate cancer combined with multiple liver metastases. For evaluating normal liver parenchyma (green), a manual segmentation was performed at the right lobe of the liver. For the liver lesion (yellow), the segmentation was completed layer by layer along the contour of the lesion. Morphological dilation was applied to segment out the peritumor (blue).

The peritumoral VOI was morphological dilated in three dimensions automatically after the segmentation of the liver tumor. Studies had shown that 3mm around hepatocellular carcinoma was the most appropriate distance to improve the prediction performance of the model (19). Therefore, 3mm was defined to expand outward as the peritumoral area, and manually removed blood vessels, hepatic ducts and the part beyond the liver parenchyma. In addition, a circular ROI with a diameter of 3.0 cm was placed on the uniform parenchyma of the right lobe of the liver at the hilar slice to evaluate the normal liver parenchyma (**Figure 3**). All delineations were completed on the same computer, and fixed window width and window level (400, 30) and all VOIs and ROIs were saved as nrrd format files.

#### 2.3.3 Radiomic feature extraction

Pyradiomics, an open-source package in 3D Slicer software, was used to extract the image radiomic features. For the same case, the delineated nrrd file was utilized for six groups of images during feature extraction, which could ensure the consistency of VOI and ROI. The extracted radiomics features included 18 first-order features (histogram), 38 second-order features (grey-level co-occurrence matrix, GLCM; grey-level difference matrix, GLDM), 37 high-order features (neighborhood grey-tone difference matrix, NGTDM; grey-level run-length matrix, GLRLM; grey-level zone-size matrix, GLZSM) and 144 (18×8 = 144) wavelet features. A total of 5688 [(18 + 38+37+144) ×4×6 = 5688] features were extracted for each patient. The 3D wavelet filters, i.e., LLH, LHL, LHH, HLL, HLH, HHL, HHH, LLL, were applied and yielded corresponding wavelet features.

### 2.4 Statistical analysis

All statistical analysis were completed with SPSS (version 25.0, IBM, Armonk, NY, USA). The Kolmogorov-Smirnov test was used for testing the normality of the data and the Levene’s test was employed to test the homogeneity of variances. In accordance with the Kolmogorov-Smirnov and Levene’s test results, One-way analysis of variance (ANOVA) or Kruskal-Wallis test were applied for comparing features among the six groups. Least Significant Difference (LSD) or Bonferroni method was used for further comparison and *P* value was adjusted using the Bonferroni correction. Intra group correlation coefficients (ICC) were used to evaluate the intra observer and inter observer consistency of the features extracted by the two radiologists. ICC < 0.40 was considered as poor consistency, 0.40 ≤ ICC ≤ 0.75 was considered as moderate consistency, and ICC > 0.75 was considered as excellent consistency. *P* < 0.05 indicated that the difference was statistically significant.

## 3 Results

### 3.1 Participants and lesion characteristics

In final, this study included 60 patients, including 41 males and 19 females, ranging from 32 to 90 years old, with an average age of (64.92 ± 12.80) years (**Table 1**). Twenty-six patients with primary liver malignant tumors, including 21 cases of hepatocellular carcinoma and 5 cases of cholangiocarcinoma, were confirmed by pathology. Thirty-nine patients with secondary liver metastasis, including 5 cases of single liver metastasis and 34 cases of multiple liver metastasis, were confirmed by pathology in 9 cases and clinical data in 30 cases. Type of metastasis: colorectal (n=14), rectal (n=6), pancreatic (n=4), esophageal (n=4), breast (n=4), pulmonary (n=2), gastric (n=2), prostate (n=2) and uterine (n=1).

**Table 1:** Participant and lesion characteristics.

Parameter	Value
No. of participants	60
Age (y)*	64.92 ± 12.80
Sex	
Male	41
Female	19
Height (cm)*	167.00 ± 7.00
Weight (kg)*	62.32 ± 10.88
Body mass index (kg/m^2^)*	22.27 ± 3.66
Liver lesions	
No. of Primary liver malignant tumors	21
No. of Hepatocellular carcinoma	17
No. of Cholangiocarcinoma	4
No. of Secondary liver metastasis	39

Unless otherwise specified, data are numbers of participants or lesions.

* Data are means ± standard deviations.

### 3.2 Radiomics analysis

#### 3.2.1 Comparison of first-order features

**Figure 4:**
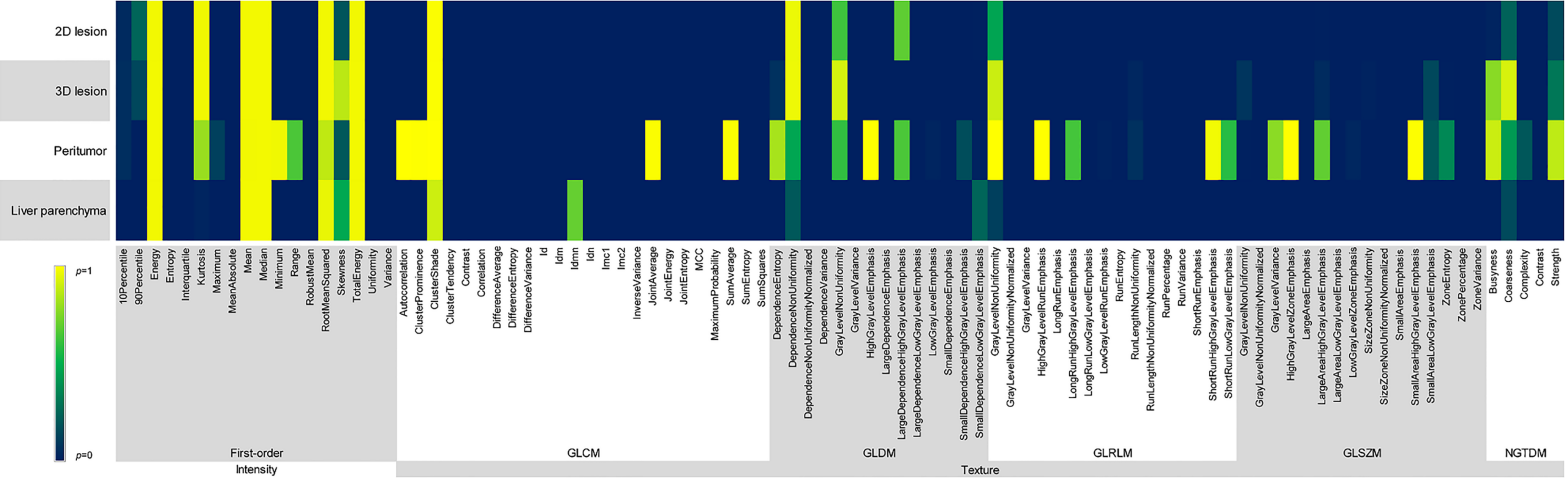
Comparison of *p* values among different reconstruction algorithms, including first-order and texture features of 2D and 3D lesions in peritumor and liver parenchyma.

**Figure 5:**
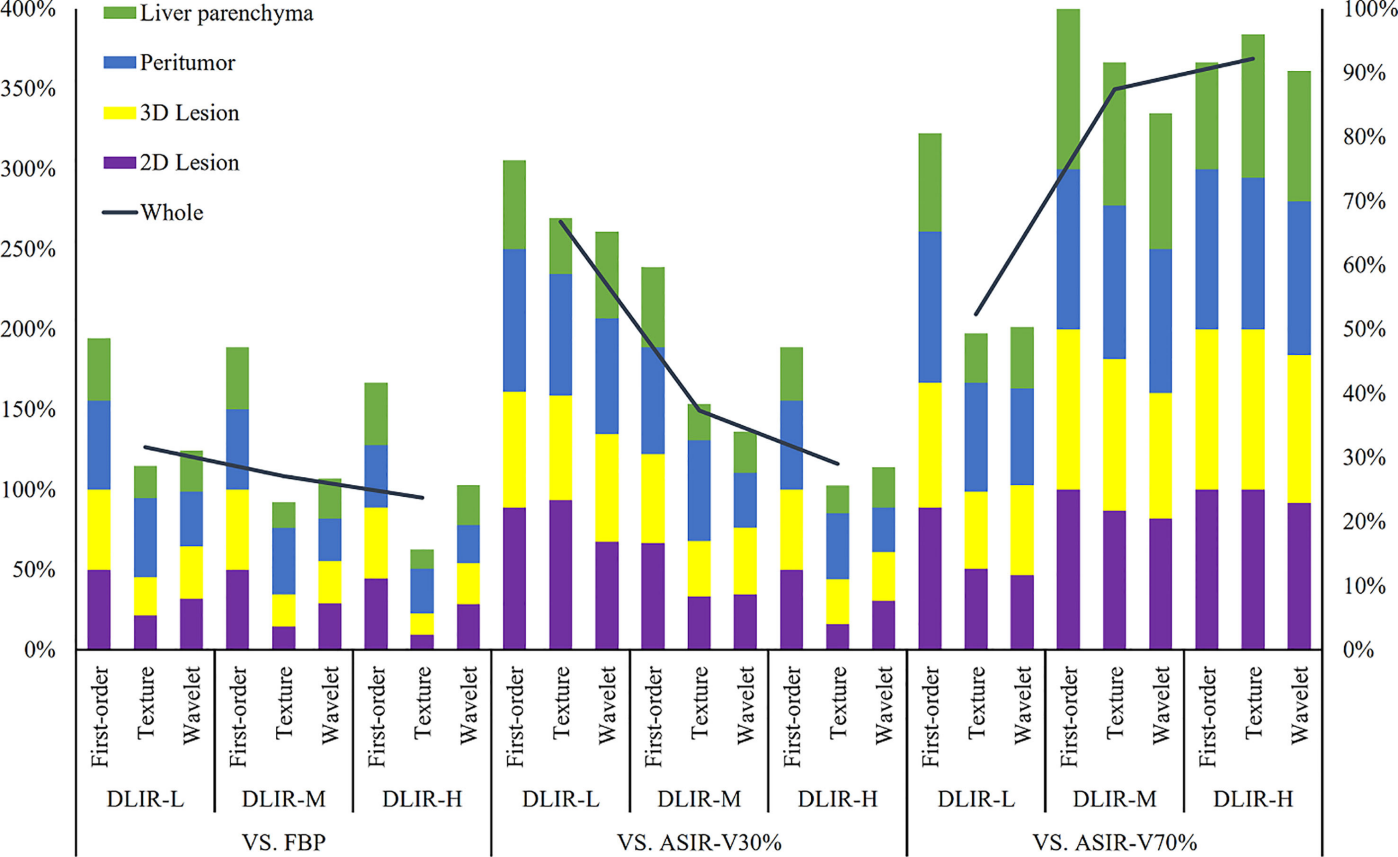
Percentages of radiomic features (including first-order, texture and wavelet) without influence in 2D lesions, 3D lesions, peritumor and liver parenchyma. The left vertical axis denoted the sum percentages of four ROIs which were illustrated on the left-top. The analysis between three levels of DLIR and FBP, ASIR-V30% and ASIR-V70% were determined according to the left vertical axis. The right vertical axis represents the sum percentages of all radiomic features of the four ROIs. FBP, filtered back projection; ASIR-V, adaptive statistical iterative reconstruction-V (ASIR-V at 30% and 70% strengths); DLIR, deep learning image reconstruction (L, low; M, medium; and H, high strengths).

Energy, Mean, Median, Root Mean Squared and Total Energy of 2D, 3D lesions, peritumor and liver parenchyma were not statistically different among six groups (*p* all > 0.05) (**Figure 4**). In addition, Skewness of 2D, 3D lesions and liver parenchyma, 90 Percentile and Kurtosis of 2D and 3D lesions were also not statistically different among groups (all *p* > 0.05). The result of pairwise comparison showed that 69.44% (44/72) (ASIR-V30% vs. FBP) and 44.44% (32/72) (ASIR-V70% vs. FBP) features had no statistical difference. The percentages of features without statistical difference were about 48.61%, 47.22%, 41.67% (DLIR-L, M, H vs. FBP), 76.39%, 59.72%, 47.22% (DLIR-L, M, H vs. ASIR-V30%), and 80.56%, 100%, 91.67% (DLIR-L, M, H vs. ASIR-V70%), as shown in **Figure 5**.

#### 3.2.2 Comparison of texture features

GLCM-Cluster Shade, GLDM-Dependence Non-Uniformity, GLRLM-Gray Level Non-Uniformity and NGTDM-Coarseness of 2D, 3D lesions, peritumor and liver parenchyma were not statistically different among six groups (all *p* > 0.05) (**Figure 4**). For texture features without statistical difference, the largest value was observed in peritumor (20/75), followed by 3D lesions (11/75), 2D lesions (7/75), and the least liver parenchyma (5/75). The results of pairwise comparison showed that 64.00% (192/576) (ASIR-V30% vs. FBP) and 17.00% (98/576) (ASIR-V70% vs. FBP) features had no statistical difference. The percentages of features without statistical difference were about 28.67%, 23.00%, 15.67% (DLIR-L, M, H vs. FBP), 67.33%, 38.33%, 25.67% (DLIR-L, M, H vs. ASIR-V30%), and 49.33%, 91.67%, 96.00% (DLIR-L, M, H vs. ASIR-V70%), as shown in **Figure 5**.

#### 3.2.3 Comparison of wavelet features

**Figure 6:**
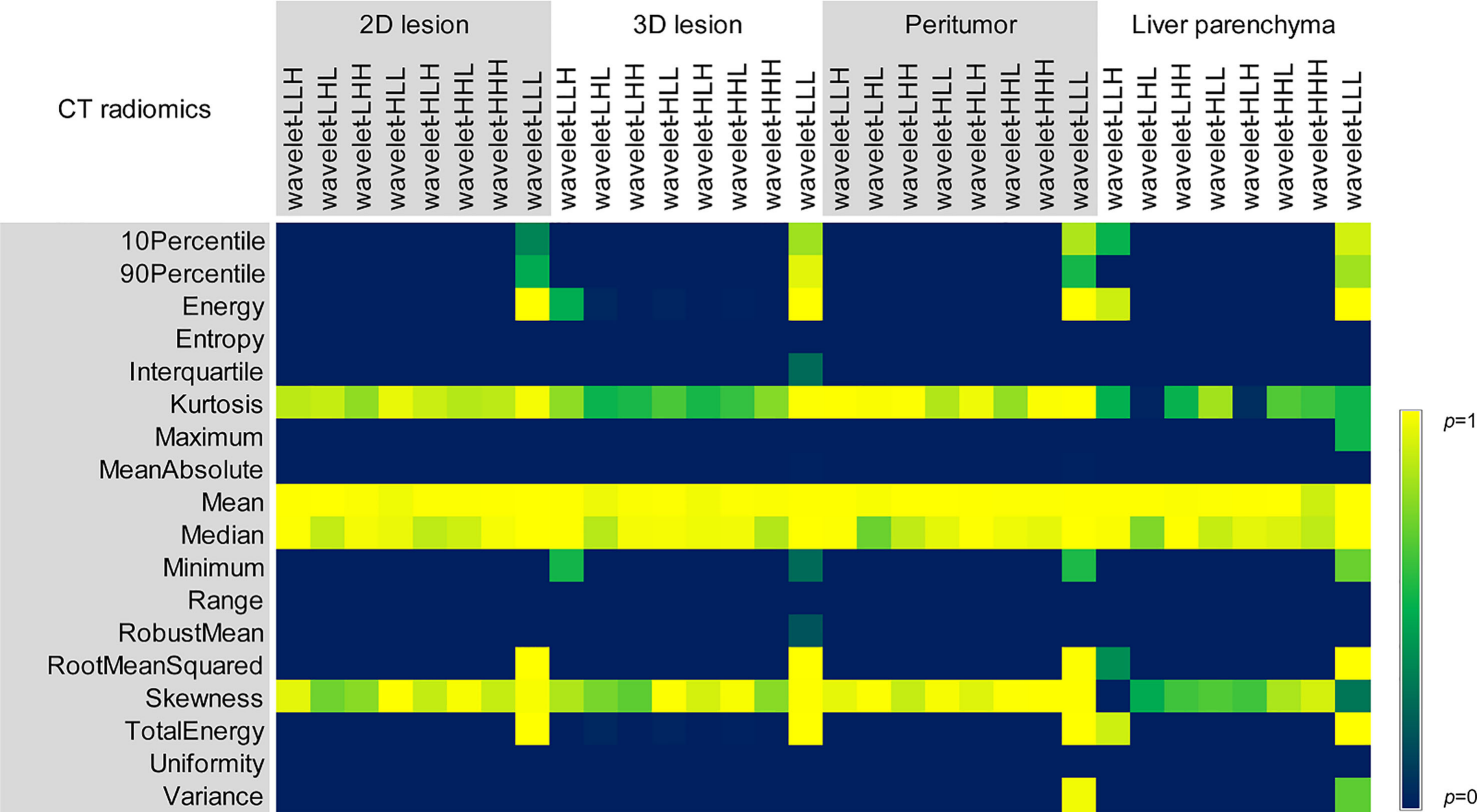
Comparison of *p* values of different reconstruction algorithms for wavelet features in 2D and 3D lesions, peritumor and liver parenchyma.

For six groups, there were no statistical differences among the Mean, Median and Skewness of 2D, 3D lesions and liver parenchyma (all *p* > 0.05). (**Figure 6**). Wavelet-LLL filtering transformation generated the largest (40/72) features without statistical difference. The results of pairwise comparison showed that 75.35% (434/576) (ASIR-V30% vs. FBP) and 25.00% (115/576) (ASIR-V70% vs. FBP) features had no statistical difference. The percentages of features without statistical difference were about 31.08%, 26.74%, 25.69% (DLIR-L, M, H vs. FBP), 65.28%, 34.03%, 28.47% (DLIR-L, M, H vs. ASIR-V30%), and 50.35%, 83.68%, 90.28% (DLIR-L, M, H vs. ASIR-V70%), as shown in **Figure 5**.

#### 3.2.4 Comparison of whole radiomic features

Different reconstruction algorithms influenced most radiomic features. The percentages of first-order, texture and wavelet features without statistical difference among 2D and 3D lesions, peritumor and liver parenchyma for all six groups were 27.78% (5/18), 5.33% (4/75) and 5.56% (1/18), respectively (all *p* > 0.05).

With the increase of ASIR-V and DLIR reconstruction strength, the number of features without statistical difference decreased. Compared with FBP, the radiomic features of ASIR-V30% and 70% without statistical difference decreased from 71.31% to 23.95%, and DLIR-L, DLIR-M, and DLIR-H decreased from 31.65% to 27.11% and 23.73%.

### 3.3 Consistency of imaging first-order feature extraction

For the extraction of first-order features of 3D liver lesions, the intra-ICC of extracted features between two measurements by radiologist A ranged from 0.898 to 0.999, and the inter-ICC between radiologist A and radiologist B diverged from 0.879 to 0.999, both with a good consistency (**Table 2**). For the extraction of texture features and wavelet features in 3D lesion, the agreements of inter- and intra-observer was inferior to those of first-order features and divergences were 0.455~1.000 and 0.421~1.000, respectively.

**Table 2:** ICC and 95% confidence interval (CI) of the two radiologists extracting first-order features of 3D lesions in FBP image.

CT radiomics	Inter-Observer		Intra-Observer	
	ICC	95% CI	ICC	95% CI
10Percentile	0.999	0.998~0.999	0.999	0.999~0.999
90Percentile	0.982	0.971~0.989	0.976	0.960~0.985
Energy	0.996	0.993~0.998	0.995	0.992~0.997
Entropy	0.959	0.932~0.975	0.939	0.900~0.963
Interquartile	0.940	0.902~0.964	0.922	0.872~0.952
Kurtosis	0.951	0.920~0.971	0.901	0.840~0.940
Maximum	0.982	0.970~0.989	0.951	0.920~0.971
Mean Absolute	0.898	0.835~0.938	0.879	0.805~0.926
Mean	0.995	0.992~0.997	0.994	0.990~0.996
Median	0.995	0.992~0.997	0.994	0.990~0.996
Minimum	0.998	0.996~0.999	0.994	0.990~0.996
Range	0.995	0.991~0.997	0.985	0.975~0.991
Robust Mean	0.953	0.922~0.972	0.938	0.898~0.962
Root Mean Squared	0.993	0.988~0.996	0.991	0.985~0.994
Skewness	0.980	0.967~0.988	0.968	0.946~0.980
Total Energy	0.996	0.993~0.998	0.995	0.992~0.997
Uniformity	0.956	0.928~0.974	0.934	0.892~0.960
Variance	0.962	0.938~0.977	0.949	0.916~0.969

ICC, Intra group correlation coefficients; CI, confidence interval.

## 4 Discussion

So far, few studies reported the impact of deep learning-based reconstruction algorithms on the extraction of radiomic features in clinical application of liver tumors (20). In this study, we showed that in liver lesions, peritumor and liver parenchyma, mean was not significantly different between groups both before and after wavelet filtering transformation, and for texture features, only four features were not significantly different between groups. In addition, for the texture features, the number of unaffected features of peritumor were more than those of liver lesions and liver parenchyma, and similar trends were found that the number of unaffected features of 3D lesions were also larger than those of 2D lesions.

In a comparative analysis of the impact of reconstruction algorithms on the radiomic features of liver lesions, Ahn et al. (21) showed that hybrid iterative reconstruction (HIR) and iterative model reconstruction (IMR) algorithms in portal venous phase images influenced the entropy, kurtosis, and skewness of 3D liver focal lesions (including hepatocellular carcinoma, cholangiocarcinoma and liver metastases). Solomon et al. (22) denoted that entropy and skewness in the first-order features of multiple lesions, including liver lesions, were affected by the iterative reconstruction (IR) algorithms, while kurtosis was unaffected. In our study, the mean, median and skewness of liver lesions were unaffected by the reconstruction algorithm regardless of the wavelet filter transform, and entropy was affected. The main reason for the difference was that the reconstruction algorithms were various and they led to different level of noise reduction which caused the alteration of CT features. The IR applied in our study preserved the spatial resolution during noise reduction, but it changed the noise texture since it caused a leftward shift of the noise power spectrum (NPS) peak (23). Conversely, other IR algorithms, such as IMR which did not use FBP data while reducing noise, generated larger differences in NPS from FBP, and medium- and high-strength iterations led to a more pronounced leftward shift of the NPS peak. Therefore, it was not surprising that the change of grayscale randomness by different noise reduction processes led to a decreased entropy (24).

In our study, DLIR exhibited a similar trend with IR. It was observed that DLIR with different reconstruction levels influenced the image histology features and the amount of affected features increased as the reconstruction level strengthened. Although DLIR maintained a similar noise texture as FBP, i.e., similar NPS peak frequency/average frequency (13), a slight left shift of NPS also had an impact on image texture (25). In addition, Yang et al. (26) showed that ASIR-V and DLIR algorithms reduced the mean value of structure similarity index (MSSIM) of the images, and MSSIM decreased with increasement of reconstruction strength and we inferred that it was the reason why radiomic features of three levels DLIR and FBP images differed. The kurtosis and skewness were sensitive to noise, and the process of noise reduction could lead to the boost of the kurtosis and skewness of the grayscale distribution. The fact that DLIR was not affected in this study also demonstrated that DLIR could maintain a certain stability of the image grayscale level during the reduction of substantial noise. While exploring the impact of reconstruction algorithms on radiomic features of 2D and 3D lesions, Prezzi et al. (16) showed that the first-order features of 2D and 3D colorectal cancer lesions had relatively small changes, while the texture features (including GLCM and GLDM) of 2D lesions had more changes. It was similar to our study that the texture features of 3D lesions were more stable than those of 2D lesions.

The parenchymal area around the tumor could reflect the microvascular invasion (MVI) of the tumor, which was valuable for the assessment of the invasive behavior of the tumor (27, 28). This study showed that different reconstruction algorithms also affect most of the peritumoral features, but in particular, the texture features of peritumor were more than those of liver lesions and liver parenchyma which were not affected. Although there were no relevant studies to explore the effect of reconstruction algorithms on peritumoral features, Tunali et al. denoted that the reproducibility of peritumoral features of lung cancer lesions was higher than that of intratumoral features (29), which echoes our findings. Peritumoral texture features could provide potential biological heterogeneity of the tumor microenvironment and better assess tumor biological behavior (30–32). A better stability of peritumoral texture features in this study also made it possible to obtain more reliable results while combining different reconstruction algorithms for CTTA. In addition, this study analyzed how the radiomic features of normal liver tissue were affected by the reconstruction algorithm. Compared with the liver lesions and peritumor, the number of unaffected features of liver parenchyma was less, which was different from the research results of Ahn et al. (21). It might be the placement and segmentation of the liver parenchyma were altered. They selected the level of the right main portal vein of the liver to manually segment the entire largest liver slice, whereas we selected a circular region of the right lobe of the liver parenchyma. Caruso et al. (33) confirmed that kurtosis and skewness in the first-order features of CT plain scanning were unaffected by algorithm (ASIR-V vs. FBP) and level (10 levels of ASIR-V) during the texture analysis of the liver parenchyma using 75 oncologic patients. This study showed that kurtosis and skewness of liver and kidney were affected by the reconstruction algorithm, while energy, mean, median and total energy of each organ tissue were not affected. These differences might be mainly caused by the intake of contrast agent, as the distribution of contrast agent among organs was different after enhancement. Chun et al. (20) showed that most radiomic features extracted from the left ventricular (LV) myocardium significantly differed (81/88) among FBP, IR and deep learning-based reconstruction (DLR) algorithms, and only mean indicated no significant difference in the first-order features. We also observed that mean, energy and total energy of liver parenchyma were not significantly affected. In addition, the slight difference might be related to the influence of noise on image grayscale distribution which could change the energy of image. Noise reduction could decrease the uniformity, but DLIR could depress corresponding influence.

It was also found that after wavelet filtering transformation, the ICCs decreases in different degrees. We speculated that the filtering transformation led to some changes in image texture. Moreover, we found that the number of unaffected features through wavelet-LLL filtering transform was larger than other wavelet filtering methods, since wavelet-LLL transform could preserve more basic structure of the image and ensure the stability of image texture to some extent among different reconstruction methods.

The present study still had some limitations. First, this study was a single-center, single-instrument study using a small study cohort. Secondly, the specific liver tumor types and tumor growth environment were not enrolled and discussed in this study. The main focus of our study was the variation of CT texture features caused by different reconstruction algorithms under the same scanning conditions, instead of the absolute or true value of each feature, thus the heterogeneity of the study population would not have an impact on our main conclusions. Finally, this study did not further explore whether the radiomic features extracted in the reconstructed images by different reconstruction algorithms had an impact on the diagnostic accuracy of liver tumor modeling and post-modeling.

In conclusion, most contrast-enhanced CT radiomic features of liver tumors, peritumor and liver parenchyma were influenced by different reconstruction algorithms and levels, and the degree of impact increased with the strengthen of DLIR reconstruction level. In fact, establishing a radiomics model based on only one reconstruction algorithm would limit the robustness of model, and selecting the subset of radiomic features which were not influenced by reconstruction algorithms could generalize the capability of model. Therefore, while conducting CTTA, we should consider the influence of reconstruction algorithm and reconstruction strength on the results to obtain more reliable results.

## Data availability statement

The original contributions presented in the study are included in the article/supplementary material. Further inquiries can be directed to the corresponding authors.

## Ethics statement

The studies involving human participants were reviewed and approved by The Qingdao Municipal Hospital Medical Ethics Committee, Qingdao Municipal Hospital. Written informed consent for participation was not required for this study in accordance with the national legislation and the institutional requirements.

## Author contributions

GX, HL, SZ, BH, and GW contributed to the conception and design. GX, XC, ZZ, and LL organized the database. HL, XC, and LL managed the patient and provided technical support. GX wrote the first draft of the manuscript. GX and XC performed the statistical analysis. SZ, BH, and GW reviewed and revised the manuscript. All authors listed have made a substantial, direct, and intellectual contribution to the work and approved it for publication. All authors contributed to the article and approved the submitted version.
